# Inhibitory Effects of Alpha-Connexin Carboxyl-Terminal Peptide on Canine Mammary Epithelial Cells: A Study on Benign and Malignant Phenotypes

**DOI:** 10.3390/cancers16040820

**Published:** 2024-02-18

**Authors:** Ivone Izabel Mackowiak da Fonseca, Marcia Kazumi Nagamine, Ayami Sato, Carlos Alberto Rossatto-Jr, Elizabeth Shinmay Yeh, Maria Lucia Zaidan Dagli

**Affiliations:** 1 Laboratory of Experimental and Comparative Oncology, School of Veterinary Medicine and Animal Science, University of São Paulo, São Paulo 05508-900, Brazil; ivone.mackowiak@gmail.com (I.I.M.d.F.); mknagamine@gmail.com (M.K.N.); sato190@toyo.jp (A.S.); carlos.rossatto@usp.br (C.A.R.-J.); 2 Institute of Life Innovation Studies, Toyo University, Tokyo 374-0193, Japan; 3 Department of Pharmacology and Toxicology, Indiana University School of Medicine, Indianapolis, IN 46202, USA; esyeh@iu.edu; 4 Melvin and Bren Simon Comprehensive Cancer Center, Indiana University School of Medicine, Indianapolis, IN 46202, USA

**Keywords:** connexin, mammary cells, canine, viability

## Abstract

**Simple Summary:**

Cell-to-cell communication is important for tissue homeostasis and is, in general, decreased in cancer cells. The αCT1 peptide mimics the regulatory cytoplasmic domain of connexin 43 (Cx43), a protein that forms the gap junctions, and redirects uncoupled Cx43 hemichannels into gap junctions, reducing gap junction turnover and changing its aggregation without affecting Cx43 protein levels. In this study, we investigated the effects of the αCT1 peptides on the viability of canine benign and malignant mammary epithelial cells. The αCT1 peptide exerted differential effects on the viability of canine adenoma and adenocarcinoma cells while preserving the normal canine mammary epithelial cells. αCT1 may be possibly included in therapeutic protocols for canine mammary neoplasms.

**Abstract:**

Mammary cancer is highly prevalent in non-castrated female dogs. Cell-to-cell communication is an important mechanism to maintain homeostasis, and connexins are proteins that assemble to form the communicating gap junctions. In many cancers, communication capacity is reduced; several approaches are being tested in order to increase the communication capacity in cancer cells and, therefore, alter their viability. This study analyzed the effects of the alpha-connexin carboxyl-terminal peptide (αCT1) on canine mammary non-neoplastic and neoplastic epithelial cells. Seven canine epithelial mammary cell lines were used. Among these, one was a normal canine epithelial mammary cell line (LOEC-NMG), two canine mammary adenomas (LOEC-MAd1 and LOEC-MAd2), and four canine mammary adenocarcinomas (LOEC-MCA1, LOEC-MCA2, LOEC-MCA3 and CF41). The αCT1 corresponds to a short Cx43 C-terminal sequence linked to an internalization sequence called the antennapedia. After 24 h of incubation, the medium containing different αCT1 peptide concentrations was added to the cells, and only the culture medium was used for control. The 3-(4,5-dimethylthiazol-2-yl)-2,5-diphenyltetrazolium bromide (MTT) test was used to quantify cell viability before treatment and 48, 72, and 96 h after the treatment. Results showed that the normal mammary epithelial cell line (LOEC-NMG) was resistant to treatment with αCT1, which is consistent with a previous study on human mammary cell lines. One of the adenoma cell lines (LOEC-MAd2) was also resistant to treatment with αCT1, although the other (LOEC-MAd1) was susceptible to treatment, mostly at 72 h after treatment. Regarding the four canine adenocarcinoma cell lines, they differ regarding the susceptibility to the treatment with αCT1. Three cell lines, canine mixed adenocarcinoma (LOEC-MCA1), canine complex adenocarcinoma (LOEC-MCA2), and commercial canine mammary adenocarcinoma cell line CF41, were susceptible to treatment with αCT1, while one canine mammary adenocarcinoma cell line (LOEC-MCA3) was resistant to treatment. In most αCT1 treated cell lines, Cx43 was strongly detected in cell membranes by immunofluorescence. We propose that αCT1 restored the cell-to-cell communication capacity of neoplastic cells and induced inhibitory effects on cell viability.

## 1. Introduction

Cancer poses a significant challenge in both human and veterinary medicine. Intensified research in veterinary oncology is essential, focusing on developing novel treatments for animal neoplasms, mirroring the efforts in human oncology.

The development of new drugs for cancer treatment goes through several stages, and the time between the discovery of an active ingredient and the launch of a new drug in the market can take a few years. Owing to the high morbidity and mortality associated with different types of cancer, the emergence of new anticancer drugs is always accompanied by great interest and expectations. There is even great pressure from patients, their families, and physicians, individually or in an organized manner, and even from the pharmaceutical industry itself, for such drugs to become available even before they are registered.

In this study, we analyzed the potential of the alpha-connexin carboxyl-terminal peptide (αCT1) to reduce the population of neoplastic cells in mammary tumors in dogs. The peptide has already been tested in vitro in human neoplasms [1] and in vivo in laboratory animals [2] and has even been used in clinical trials in humans [3]. We analyzed whether αCT1 peptide had the same effect on animal neoplasms. Therefore, we tested it in primary cultures of canine mammary normal and neoplastic cells.

Under typical circumstances, the mammary gland comprises various cell types that interact not only with each other but also with other components of the tissue microenvironment, including neighboring mesenchymal cells and the extracellular matrix. These interactions involve physical and dynamic organization facilitated by distant signals, growth factors, signaling molecules, and tight junctions. Tight junctions, facilitated by proteins like occludin and zonula occludens, play a crucial role in mediating these interactions [4], gap junctions [5], cadherin-mediated adhesion junctions [6], and desmosomes [7]. Many of these junctions are interdependent and likely regulated through a coordinated process [8].

Early changes associated with carcinogenesis include alterations in gap-junctional intercellular communication (GJIC), which can suppress tumor cell growth upon junction restoration [9,10,11,12,13,14]. Furthermore, activation of protein kinases by phorbol ester and epidermal growth factor downregulates GJIC [15,16,17,18,19]. GJIC has been implicated in various biological functions, including the regulation of cell growth and differentiation [20].

Gap junctions serve as specialized membrane channels that enable intercellular communication by facilitating the exchange of ions, second messengers, and small metabolites (typically <1 kDa in size) between neighboring cells. They also play a role in electrical propagation in excitable tissues [21]. The core protein components of the gap junction channel are connexins (CXS), which are tetraspan transmembrane proteins comprising two extracellular loops, a cytoplasmic loop, and N-terminal and C-terminal cytoplasmic domains. Six connexins assemble to form transmembrane tubular hemichannels, which then couple with hemichannels in adjacent cells to create intercellular channels. These intercellular channels aggregate to form gap junctions [22,23].

The human genome contains 21 connexin genes, each exhibiting a unique tissue- and cell-type expression pattern. Gap junctions are widely acknowledged for their involvement in tumorigenesis, metastatic disease progression, and in vivo tumor proliferation [24]. Several studies have implicated Cx43 in mammary gland development [25,26,27,28,29,30,31], and Cx43 expression has been investigated in breast cancer cells or human breast cancer tissues [25,32,33,34,35,36,37,38,39,40,41].

These studies indicate that Cx43 influences the proliferation, differentiation, and migration of breast cancer cells. Additionally, investigations on human breast cancer tissues reveal alterations in Cx43 expression patterns across different cancer stages [35,36,37,38]. It is also proposed that reduced Cx43 levels and altered gap junction localization might correlate with disease severity, independent of Cx43 expression levels, implying that the absence of Cx43 at gap junctions could serve as a malignancy biomarker. Consequently, the maintenance of Cx43 function in gap junctions, in addition to preserving the communication deficit at intercellular junctions, has the potential to attenuate malignant transformation and metastatic progression [35,36,39].

Unfortunately, targeting Cx43 in breast cancer with therapeutic interventions presents challenges because connexins have diverse functions during tumor cell spread. In the early stages of malignant progression in breast and neoplastic tissue, there is a deficit in intercellular junction communication, likely related to alterations in cell–cell adhesion. Evidence shows that the loss of adherent junctions allows cells to physically separate, facilitating invasion and metastatic disease progression [24,42,43,44]. However, reports also indicate upregulation of Cx43 in established metastatic breast cancer lesions, suggesting connexins may be involved in later stages of metastasis, including tissue extravasation and colonization [32,37,45,46].

Additionally, during cancer progression, Cx43 expression has been observed to relocate to stromal compartments, indicating its potential role in regulating invasion and metastasis through interactions between epithelial tumor cells and stroma [47]. It is important to note that conflicting data likely stem from differences in experimental approaches, the cellular heterogeneity of tumors, overlapping functions of other connexin family members, and the complexity of the metastatic process.

The role of Cx43 in breast cancer likely involves channel-independent functions, and its diverse activities may be relevant. To elucidate Cx43’s role in breast cancer and develop targeted therapeutic drugs, it is crucial to examine the endogenous activity of Cx43 in gap junctions. However, studies investigating Cx43 in breast and breast tumor cells often utilize drugs that globally inhibit gap junction activity, affecting not only Cx43 but also other connexins, or employ constructs altering mRNA and Cx43 protein expression levels. Consequently, this introduces another variable into the interpretation. [26,28,48,49,50,51,52,53].

To analyze endogenous Cx43 activity at gap junctions without modifying expression levels, Rhett et al. [54] used a distinctive 25-amino-acid peptide (αCT1) designed to mimic the regulatory cytoplasmic domain of Cx43. This peptide redirects uncoupled Cx43 hemichannels into gap junctions, thereby reducing gap junction turnover and altering gap junction aggregation without impacting Cx43 protein levels [54,55]. Consequently, αCT1 provides the desired characteristics, as we were able to separate the expression levels of Cx43 from its function of Cx43. This enabled us to investigate Cx43 activity at the gap junction (communication) in breast cancer. Grek et al. [1] provided evidence showing that augmentation of Cx43 composite gap junctions by αCT1 impedes proliferation or triggers apoptosis in breast cancer cells while having no such effect on untransformed mammary epithelial cells. This confirmed the tumor suppressor role of Cx43 in breast cancer.

Furthermore, the junctional stabilization of Cx43 and modulation of intercellular communication by gap junctions in cancer cells have provoked a “bystander” effect, where an increase or decrease in the number or size of intercellular communication junctions intersperses an increase in the diffusion of cytotoxic agents and sensitization to chemotherapeutic agents in addition to the amplification of the therapeutic response [24,56]. Supporting this claim, studies on Cx43 re-expression have demonstrated an increase in the sensitivity of human cancer cells to common chemotherapeutic agents [57,58].

Grek et al. [1] provided evidence that the αCT1 connexin mimetic peptide modulates Cx43 signaling, enhancing the opening of intercellular communication junctions in breast cancer cells. They demonstrated its effectiveness in increasing drug-induced cytotoxicity in specific tamoxifen therapies for estrogen receptor (ER) in MCF7 breast cancer cell lines and lapatinib therapy for HER2 in BT474 breast cancer cell lines. Lapatinib is known to induce HER2 stabilization [59]. In addition, the same authors [1] demonstrated that the αCT1 peptide was effective in modulating the junctional activity and distribution of Cx43 in breast cancer cells and highlighted the therapeutic potential of the αCT1 peptide in breast cancer treatment.

Previous studies have established that the mechanism of action for αCT1 is disrupting the Cx43 interaction with the zonula occludens junction protein 1 (ZO-1) [54,55] because ZO-1 downregulates Cx43 and prevents gap junction formation.

Our group evaluated the role of the αCT1 peptide in canine melanoma cell lines and observed that the Bowman–Birk inhibitor increased the αCT1 growth inhibitory effects [60]. This study aimed to evaluate the in vitro effects of α-connexin carboxyl-terminal peptide (αCT1) on the viability of normal and neoplastic canine mammary tumor cells.

## 2. Materials and Methods

### 2.1. Ethics

This study was approved by the Committee on Ethics for the Use of Animals of the School of Veterinary Medicine and Animal Science of the University of Sao Paulo, Brazil (CEUAx No. 4775130918)**.**

### 2.2. Cell Lines

Seven canine mammary cell lines were used in this study. These cells (except CF41.mg) were isolated and established at the Laboratory of Experimental and Comparative Oncology of the School of Veterinary Medicine and Animal Science of the University of Sao Paulo and characterized as described by Gentile et al. [61]. Among these, one was a normal epithelial mammary cell line, two were mammary adenomas, and three were adenocarcinomas (Table 1).

### 2.3. αCT1 Peptide

The therapeutic α-connexin carboxyl-terminal peptide (αCT1) and its reverse sequence peptide (R-PEP) were synthesized by the American Peptide Company (Sunnyvale, CA, USA). The αCT1 peptide corresponds to a short Cx43 C-terminal sequence linked to an internalization sequence called antennapedia (RQPKIWFPNRRKPWKKRPRPDDLEI), as described and characterized by Hunter et al. (2005). The antennapedia-internalizing peptide sequence was RQPKIWFPNRRKPWKK.

The R-PEP sequence (RQPKIWFPNRRKPWKKRPSSRASSRASSRPRPDDLEI) consists of a reverse sequence of αCT1 linked to the antennapedia-internalizing sequence. The peptides were purchased from FirstString Research, Inc. (Mount Pleasant, SC, USA).

### 2.4. Experimental Procedures

For this assay, cells were cultured in T75 bottles with their respective media (Table 2). After cell confluence, the culture medium was discarded, and the cells were washed with 3 mL of phosphate-buffered saline (PBS) (Gibco Invitrogen, Carlsbad CA, USA), trypsinized with 1000 µL of trypsin (Gibco Invitrogen, Carlsbad CA, USA), and incubated at 37 °C for about 15 min and then inactivated with 5 mL of DMEM medium (10% fetal bovine serum (FBS) −1% penicillin/streptomycin). The supernatant was collected and centrifuged at 1500× *g* rpm for 5 min. The formed cell pellet was resuspended in fresh medium, and 10 µL of the cell suspension was collected and homogenized with 10 µL of Trypan blue for counting in the Neubauer chamber. The cell density was calculated for the respective uniform distribution of cells in a 96-well plate. For the CF41.mg line, it was calculated at 0.3 × 10^4^ cells per well, and for the other canine lines, 0.5 × 10^4^ cells per well. Furthermore, 187.5 µL of medium containing the respective strains was added to each well of the culture plate.

After 24 h of incubation, 62.5 µL of the respective medium containing high αCT1 peptide concentrations (25, 50, 100, 150, 200, 250, and 300 µM (4× concentrates)) was added, and each dose was represented in triplicate on the plate. CF41.mg, LOEC-NMG, LOEC-MCA1, LOEC-MCA2, LOEC-MCA3, LOEC-MAd1, and LOEC-MAd2 cell lines were treated for 48, 72, and 96 h. For control, cells with the culture medium only were used. MTT solution was added to the culture medium (10 µL of MTT/100 µL of medium). The cells were incubated for 3 h at 37 °C in a CO_2_ incubator and protected from light. Then, the plates were centrifuged at 4000× *g* rpm for 10 min, and the supernatant was discarded. Moreover, 100 µL/well of DMSO was added to solubilize formazan crystals. The absorbance was measured at 570 nm using a microplate reader. For the blank, complete medium containing MTT was used.

### 2.5. Immunofluorescence for Cx 43

The six LOEC cell lines (LOEC-NMG, LOEC-MCA1, LOEC-MCA2, LOEC-MCA3, LOEC-MAd1, and LOEC-MAd2) were grown on a coverslip in six-well plates in advanced DMEM, HEPES (10 mM), L-glutamine (2 mM), 1% antibiotic–antimycotic, cholera toxin (10 ng/mL), 1% mammary epithelial growth supplement), 0.5% FBS, 100 units/mL penicillin G, and 100 mg/mL streptomycin at 37 °C in a humidified atmosphere of 5% CO_2_. Only the CF41.mg line was cultivated with DMEM, 10% fetal bovine serum, and L-glutamine (2 mM). After 2 days of cultivation, the cells were washed with PBS and fixed in 70% ethanol for 20 min at −20 °C. Cells were permeabilized with PBS containing 0.1% Triton X-100 for 20 min. Then, the coverslips were subjected to antigen exposure for 30 min in boiling with ethylenediaminetetraacetic acid (EDTA) at pH 9.0.

Coverslips were incubated in EDTA until they were completely cooled. Then, the coverslips were washed and incubated in a humid chamber overnight at 4 °C with monoclonal anti-connexin 43 antibody (Zymed Laboratories Inc, San Francisco, CA, USA catalog number- 71.0700).

The next day, the coverslips were washed with PBS containing 0.1% Triton X-100 and incubated with secondary antibodies labeled with Alexa 488 diluted 1:100 (Abcam, Cambridge, UK) for 60 min in a humid chamber. The coverslips were washed with PBS containing 0.1% Triton X-100 and exposed for 20 min to a solution containing propidium iodide, washed, and mounted with Vectashield without DAPI to prevent fluorescence depletion (Vector Laboratories, Inc., Burlingame, CA, USA), and the coverslips were sealed with enamel.

### 2.6. Statistical Analysis

Statistical analysis of the results was performed using one-way analysis of variance (ANOVA), followed by Dunnett’s test, with a significance level of *p* < 0.05.

## 3. Results

### 3.1. Canine Mammary Cell Lines, Their Shapes, and Growth Patterns

**Figure 1:**
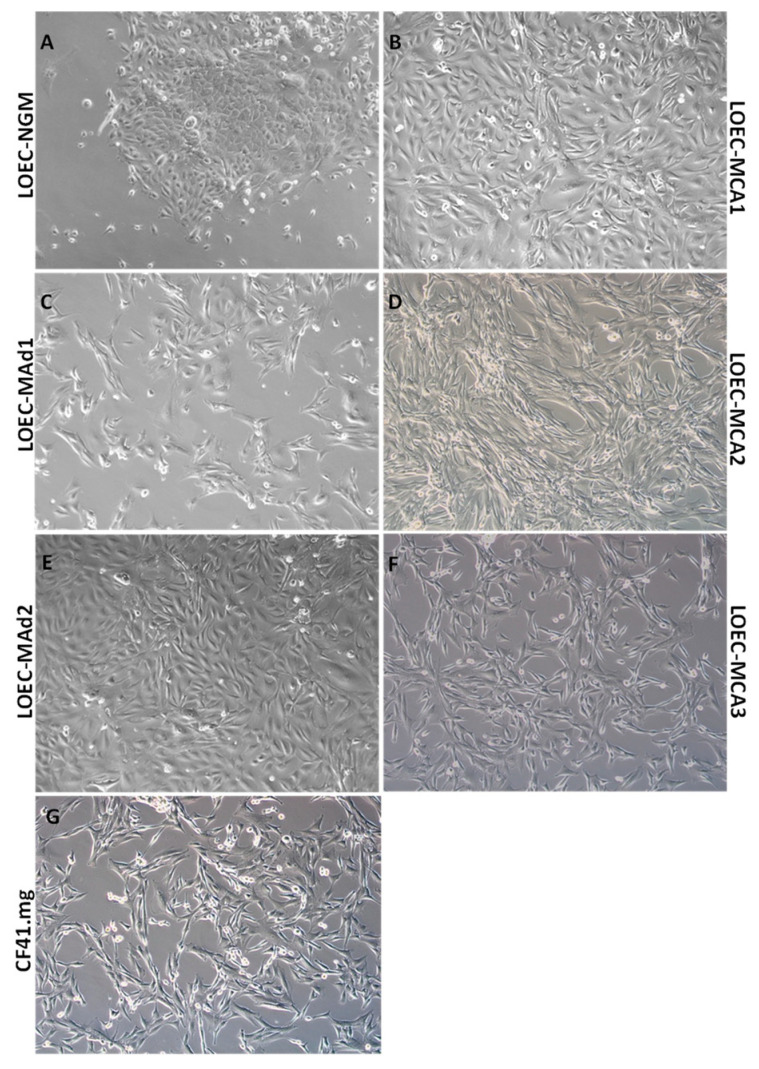
Bright-field photomicrographs of the 7 canine mammary cell lines used in this study (**A–G**); 40× objective.

Figure 1 shows the bright-field images of the seven canine mammary cell lines used in this study. It is possible to notice some differences in their shapes and growth patterns.

The LOEC-NGM (Figure 1A), which are normal canine mammary cells, have an epithelial shape and grow more slowly when compared to the neoplastic cell lines. LOEC-MAd1 (Figure 1C), a mixed adenoma, has a predominantly spindle shape, and some larger epithelioid cells are also seen. LOEC-MAd2 (Figure 1E), a myoepithelial adenoma cell line, also has a predominantly epithelioid shape; these cells grow faster than LOEC-MAd1, doubling the number of cells in a few hours. Additionally, these cells detach very easily during trypsinization.

The four canine mammary adenocarcinoma cells used in this study share some characteristics. LOEC-MCA1 (Figure 1B) is derived from a mixed adenocarcinoma; it is composed of cells with both epithelial and spindle shapes. They present a different growth pattern when compared to the adenomas. The LOEC-MCA2 (Figure 1D) is derived from a complex adenocarcinoma and has a spindle appearance. It has accelerated growth, forming layers of overlapping cells. The LOEC-MCA3 (Figure 1F), derived from a mammary adenocarcinoma, also has a spindle appearance and accelerated growth, forming layers of overlapping cells. The CF41.mg lineage (Figure 1G), derived from a mammary adenocarcinoma, shows a marked spindle pattern, the same characteristics of accelerated growth, doubling the number of cells in 24 h of culture.

### 3.2. Viability of the LOEC- NMG Canine Mammary Epithelial Cell Line after Treatment with αCT1 Peptide

**Figure 2:**
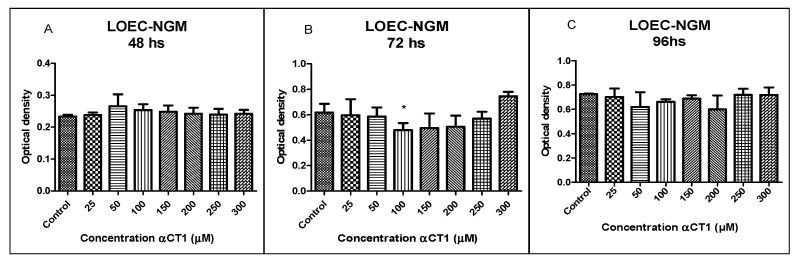
Viability of LOEC-NMG canine mammary lineage at 48, 72, and 96 h after the treatment with αCT1 peptide. No alterations in cell viability were seen when compared with the control, except at 72 h, 100 µM. (**A**). LOEC-NMG treated with αCT1 peptide for 48 h showed no statistical differences when compared to control; (**B**). LOEC-NMG treated with αCT1 peptide for 72 h showed a significant decrease in viability when compared to control, only at the concentration of 100 µM; (**C**). LOEC-NMG treated with αCT1 peptide for 96 h showed no statistical differences when compared to control (one-way ANOVA statistical analysis * *p* < 0.05).

Treatment with different αCT1 peptide concentrations (25, 50, 100, 150, 200, 250, and 300 µM) in the LOEC-NMG strain did not show an inhibitory activity in any of the concentrations at 48 and 96 h (Figure 2). The 72 h treatment showed inhibitory activity, with a statistical difference of * *p* < 0.05 at a concentration of 100 µM.

### 3.3. Viability of the LOEC-MAd1 Canine Mammary Epithelial Cell Line after Treatment with αCT1 Peptide

**Figure 3:**
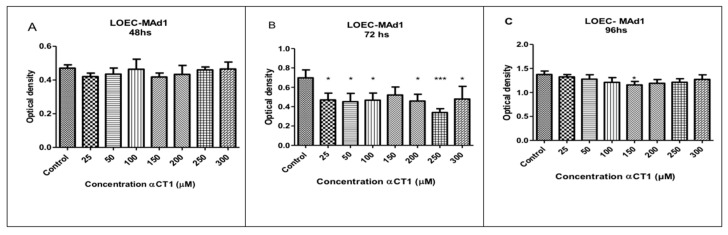
Viability of LOEC-MAd1 canine mammary adenoma lineage at 48, 72, and 96 h after the treatment with αCT1 peptide. Significant decreases in viability were observed at 72 h in LOEC-MAd1 (**A**). LOEC- MAd1 treated with αCT1 peptide for 48 h showed no statistical differences when compared to control; (**B**). LOEC- MAd1 treated with αCT1 peptide for 72 h showed statistically significant decreases in viability when compared to control at all concentrations (from 25 μM to 300 μM); (**C**). LOEC- MAd1 treated with αCT1 peptide for 96 h showed no statistical differences when compared to control. (one-way ANOVA statistical analysis (* *p* < 0.05, *** *p* < 0.0001).

The treatment with different αCT1 peptide concentrations (25, 50, 100, 150, 200, 250, and 300 µM) in the LOEC-MAd1 strain did not show an inhibitory activity in any of the concentrations at the time of 48 h. However, the 72 h treatment showed significant inhibitory activity of * *p* < 0.05 at concentrations of 25, 50, 100, 200, and 300 µM and *** *p* < 0.0001 at a concentration of 250 µM (Figure 3). The LOEC-MAd1 strain showed a time sensitivity of 96 h with a statistical difference of * *p* < 0.05 at a concentration of 150 µM.

### 3.4. Viability of the LOEC-MAd2 Canine Mammary Epithelial Cell Line after Treatment with αCT1 Peptide

**Figure 4:**
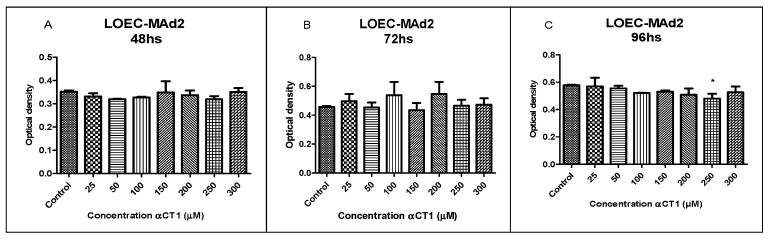
Viability of LOEC-Mad2 canine mammary adenoma lineage at 48, 72, and 96 h after the treatment with αCT1 peptide. A significant decrease in viability was observed at 96 h in LOEC-MAd2. (**A**). LOEC- MAd2 treated with αCT1 peptide for 48 h showed no statistical differences when compared to control; (**B**). LOEC- MAd2 treated with αCT1 peptide for 72 h showed no statistical differences when compared to control; (**C**). LOEC- MAd1 treated with αCT1 peptide for 96 h showed a statistically significant decrease in viability when compared to control only at the concentration of 250 µM (one-way ANOVA statistical analysis (* *p* < 0.05).

The results of treatment with different αCT1 peptide concentrations (25, 50, 100, 150, 200, 250, and 300 µM) in the LOEC-MAd2 strain did not show inhibitory activity, with a statistical difference at any of the concentrations at 48 and 72 h. The 96 h treatment showed inhibitory activity, with a statistical difference of * *p* < 0.05 at a concentration of 250 µM (Figure 4).

### 3.5. Viability of the LOEC-MCA1 Canine Mammary Cell Line after Treatment with αCT1 Peptide

**Figure 5:**
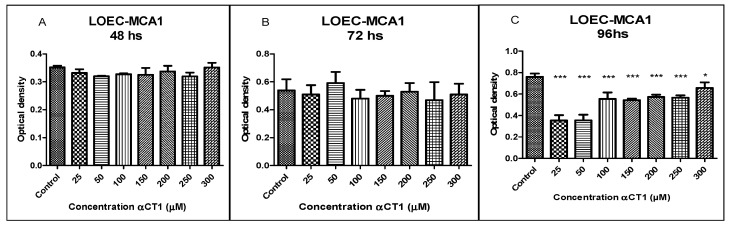
Viability of LOEC-MCA1 canine mammary adenocarcinoma lineage at 48, 72, and 96 h after the treatment with αCT1 peptide. One-way ANOVA statistical analysis for the LOEC-MCA1 canine mammary lineage showed a significant difference at 96 h treatment. (**A**). LOEC- MCA1 treated with αCT1 peptide for 48 h showed no statistical differences when compared to control; (**B**). LOEC- MCA1 treated with αCT1 peptide for 72 h showed no statistical differences when compared to control; (**C**). LOEC- MCA1 treated with αCT1 peptide for 96 h showed statistically significant decreases in cell viability when compared to control for all αCT1 peptide concentrations (* *p* < 0.05, *** *p* < 0.0001).

The treatment of the LOEC-MCA1 strain with different αCT1 peptide concentrations (50, 100, 150, 200, 250, and 300 µM) did not present statistically significant inhibitory activity at any of the concentrations at 48 and 72 h (Figure 5). However, at 96 h of treatment, the LOEC-MCA1 strain showed sensitivity to all peptide concentrations with a statistical difference of *** *p* < 0.001 for concentrations of 25, 50, 100, 150, 200, and 250 µM and * *p* < 0.05 for the concentration of 300 µM.

### 3.6. Viability of the LOEC-MCA2 Canine Mammary Cell Line after Treatment with αCT1 Peptide

**Figure 6:**
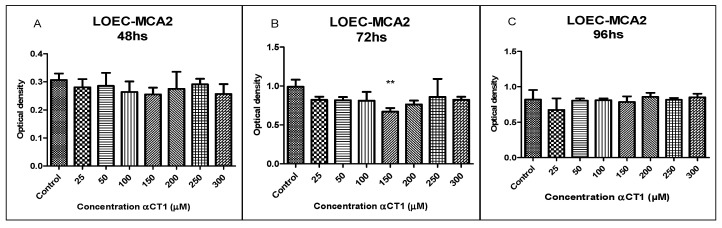
Viability of LOEC-MCA2 canine mammary adenocarcinoma lineage at 48, 72, and 96 h after the treatment with αCT1 peptide. One-way ANOVA statistical analysis for the LOEC-MCA2 canine mammary lineage showed a significant decrease in viability at 72 h of treatment, with a concentration of 150 µM. (**A**). LOEC- MCA2 treated with αCT1 peptide for 48 h showed no statistical differences when compared to control; (**B**). LOEC- MCA2 treated with αCT1 peptide for 72 h showed a statistically significant decrease in viability when compared to control only at the concentration of 150 µM; (**C**). LOEC- MCA2 treated with αCT1 peptide for 96 h showed no statistical differences when compared to control (** *p* < 0.01).

The treatment with different αCT1 peptide concentrations (25, 50, 100, 150, 200, 250, and 300 µM) in the LOEC-MCA2 cell line showed no statistically different inhibitory activity in any of the concentrations at 48 and 96 h, demonstrating increased resistance to treatment with the peptide (Figure 6). The LOEC-MCA2 strain showed sensitivity after 72 h of treatment at an αCT1 peptide concentration of 150 µM with a statistical difference of ** *p* < 0.01.

### 3.7. Viability of the LOEC-MCA3 Canine Mammary Cell Line after Treatment with αCT1 Peptide

**Figure 7:**
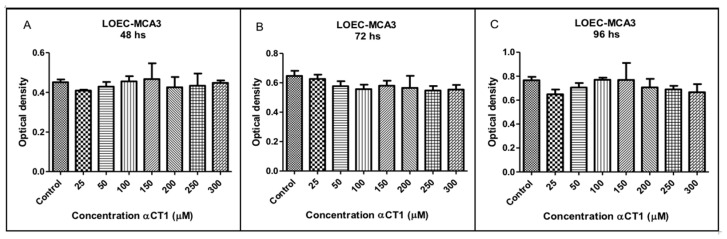
Viability of LOEC-MCA3 canine mammary adenocarcinoma lineage at 48, 72, and 96 h after the treatment with αCT1 peptide. One-way ANOVA statistical analysis for the LOEC-MCA3 canine mammary lineage has shown no statistical differences in comparison with controls. (**A**). LOEC- MCA3 treated with αCT1 peptide for 48 h showed no statistical differences when compared to control; (**B**). LOEC- MCA3 treated with αCT1 peptide for 72 h showed no statistical differences when compared to control; (**C**). LOEC- MCA3 treated with αCT1 peptide for 96 h showed no statistical differences when compared to control.

The treatment with different αCT1 peptide concentrations (25, 50, 100, 150, 200, 250, and 300 µM) in the LOEC-MCA3 strain did not show any inhibitory activity, with no statistical difference at any of the concentrations at 48, 72, and 96 h (Figure 7).

### 3.8. Viability of the CF41.mg Canine Mammary Cell Line after Treatment with αCT1 Peptide

**Figure 8:**
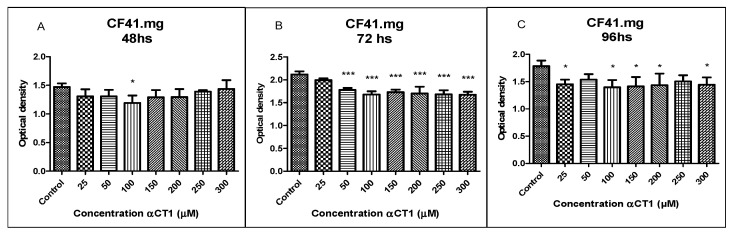
Viability of CF41.mg cells after treatment with αCT1 peptide. The αCT1 peptide treatments were compared with the control. One-way ANOVA statistical analysis was used. Cells were highly sensitive to treatment. (**A**). CF41.mg cells treated with αCT1 peptide for 48 h showed a statistically significant decrease in optical density (cell viability) when compared to control only at 100 µM; (**B**). CF41.mg cells treated with αCT1 peptide for 72 h showed statistically significant decreases in optical density (cell viability) when compared to control at all concentrations of αCT1 peptide; (**C**). CF41.mg cells treated with αCT1 peptide for 96 h showed statistically significant decreases in optical density (cell viability) when compared to control at the concentrations of 25, 100, 150, 200 and 300 µM of the αCT1 peptide. (* *p* < 0.05 and *** *p* < 0.0001).

The results of the treatment with αCT1 peptide revealed an inhibitory activity with a statistical difference of * *p* < 0.05 at a concentration of 100 µM at 48 h in the CF41.mg strain. After 72 h of treatment with the same strain, inhibitory activity was observed with a statistical difference of *** *p* < 0.001 at αCT1 concentrations of 50, 100, 150, 200, 250, and 300 µM (Figure 8B). At 96 h of treatment, CF41.mg had a slightly lower sensitivity than at 72 h, with a statistical difference of * *p* < 0.05 at concentrations of 25, 100, 150, 200, and 300 µM (Figure 8).

### 3.9. Summary of the Results

Table 3 shows a summary of the results of treatment with αCT1 peptide on canine mammary cell lines. At 48 h after treatment, only CF41 cells showed a significant reduction in cell viability at the concentration of 100 µM. At 72 h after treatment, the CF41.mg strain showed a significant reduction in cell viability at almost all αCT1 concentrations, and LOEC-MAd1 presented significant reductions at almost all concentrations except 150 µM. At 96 h, LOEC-MCA1 showed a high statistical difference in viability when compared with controls at all concentrations of αCT1, and CF41.mg also showed decreased viability when compared to controls.

### 3.10. Expression and Subcellular Localization of Cx43 in Canine Mammary Cell Lines after TREATMENT with αCT1

**Figure 9:**
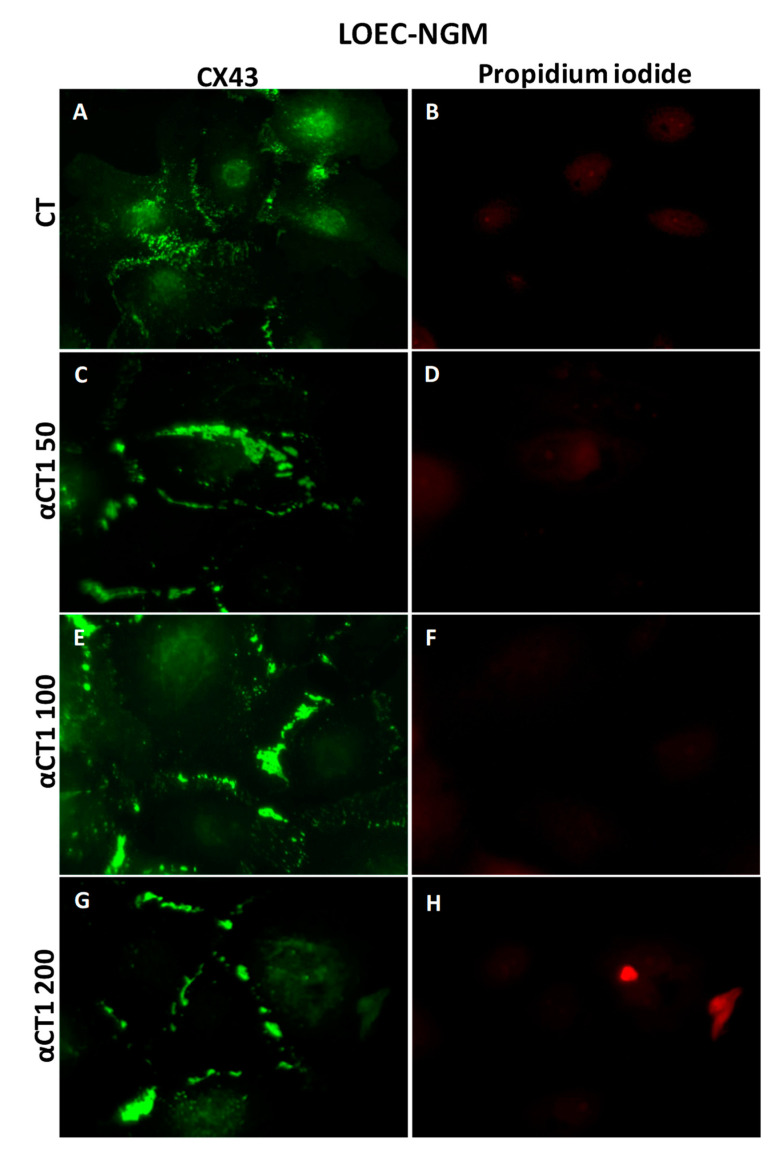
Immunofluorescence photomicrographs for CX43 in the canine normal mammary gland lineage LOEC-NMG. In (**E**,**G**), strong positivity of Cx43 in cell membranes can be observed; (**A**,**C**,**E**,**G**) show immunofluorescence positivity of Cx43 in cell membranes; (**B**,**D**,**F**,**H**) show cell nuclei stained with propidium iodide. 40× objective.

The Cx43 expression profiles in canine mammary strains exposed to αCT1 treatment were evaluated and compared using an immunofluorescence assay. Immunofluorescence results showed that the LOEC-NGM cells presented Cx43 located in the membranes, and the treatment with αCT1 peptide increased the Cx43 plaques in cell membranes (Figure 9).

**Figure 10:**
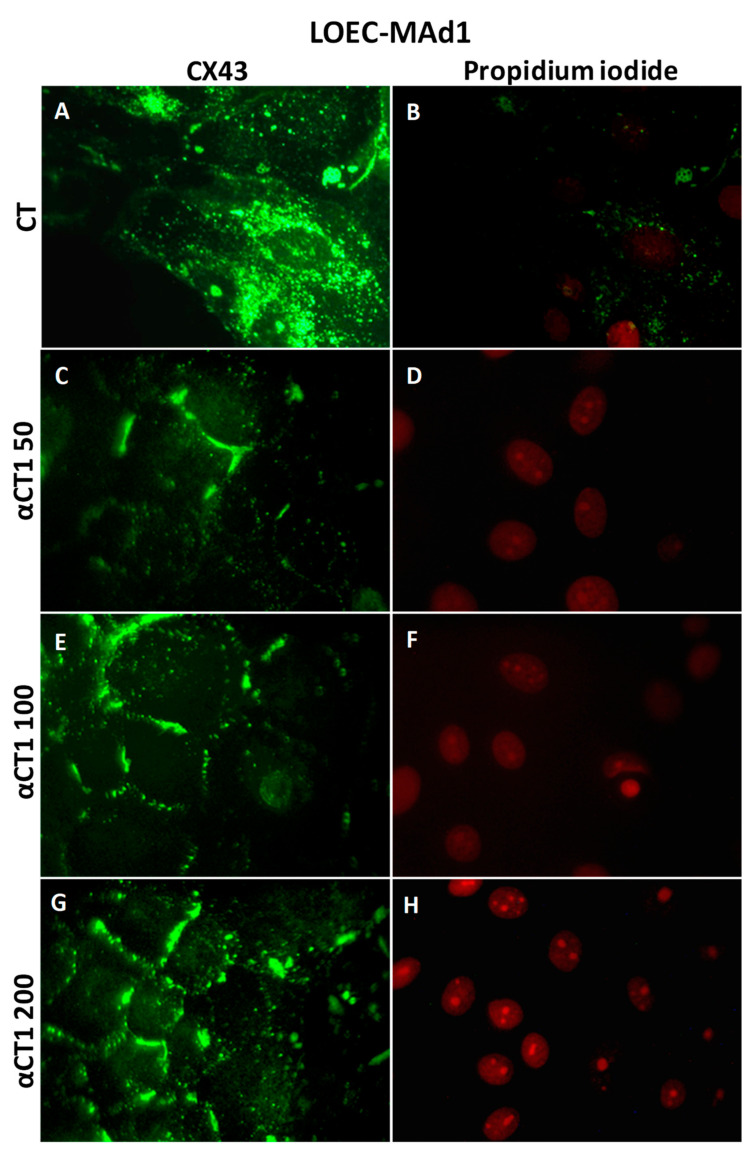
Immunofluorescence photomicrograph for CX43 in the canine mammary adenoma lineage LOEC-MAd1. In (**E**,**G**), strong positivity of Cx43 in cell membranes can be observed; (**A**,**C**,**E**,**G**) show immunofluorescence positivity of Cx43 in cell membranes; (**B**,**D**,**F**,**H**) show cell nuclei stained with propidium iodide. 40× objective.

**Figure 11:**
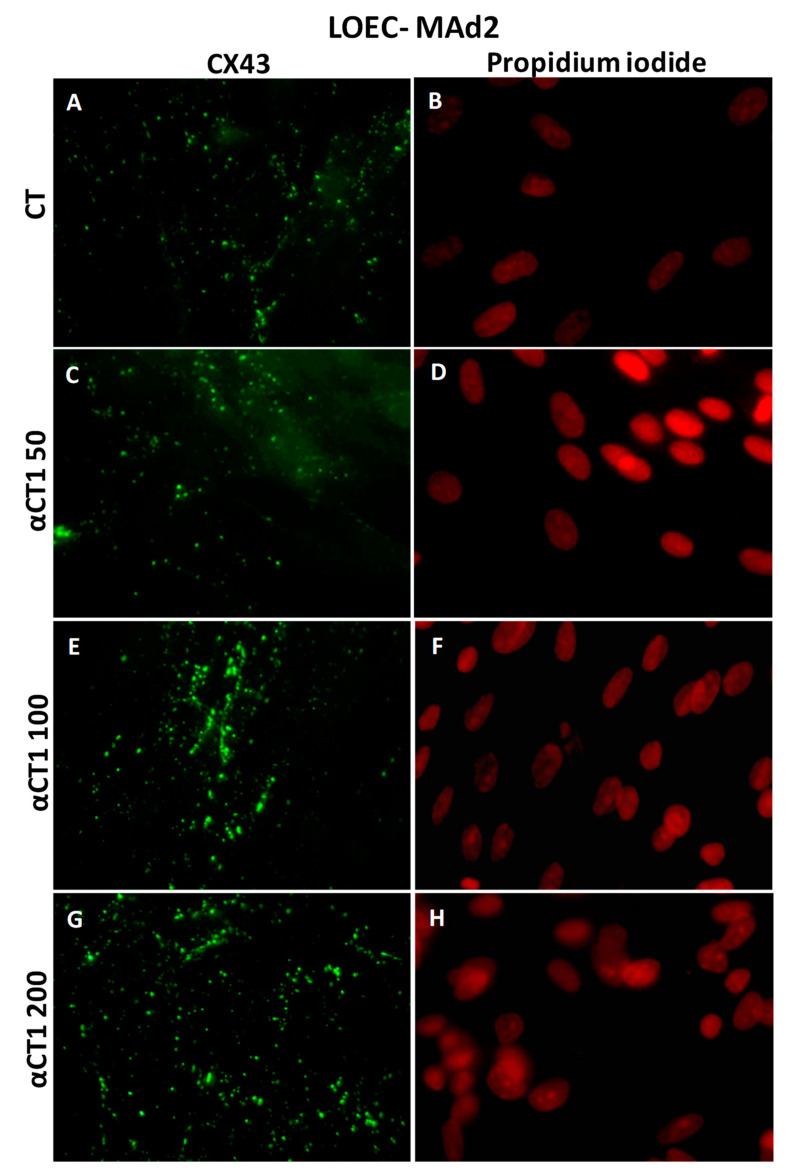
Immunofluorescence photomicrograph for CX43 in the canine mammary adenoma lineage LOEC-MAd2. In (**G**), strong positivity of Cx43 in cell membranes can be observed; (**A**,**C**,**E**,**G**) show immunofluorescence positivity of Cx43 in cell membranes; (**B**,**D**,**F**,**H**) show cell nuclei stained with propidium iodide 40× objective.

Figure 10 shows that LOEC-MAd1 control cells presented the expression of Cx43 in the cytoplasm but also in the cell membranes; the αCT1 peptide increased the Cx43 plaques in cell membranes. LOEC MAd2 also presented Cx43 localized in both cytoplasm and membranes and the treatment with the αCT1 peptide increased the Cx43 plaques in cell membranes (Figure 11).

**Figure 12:**
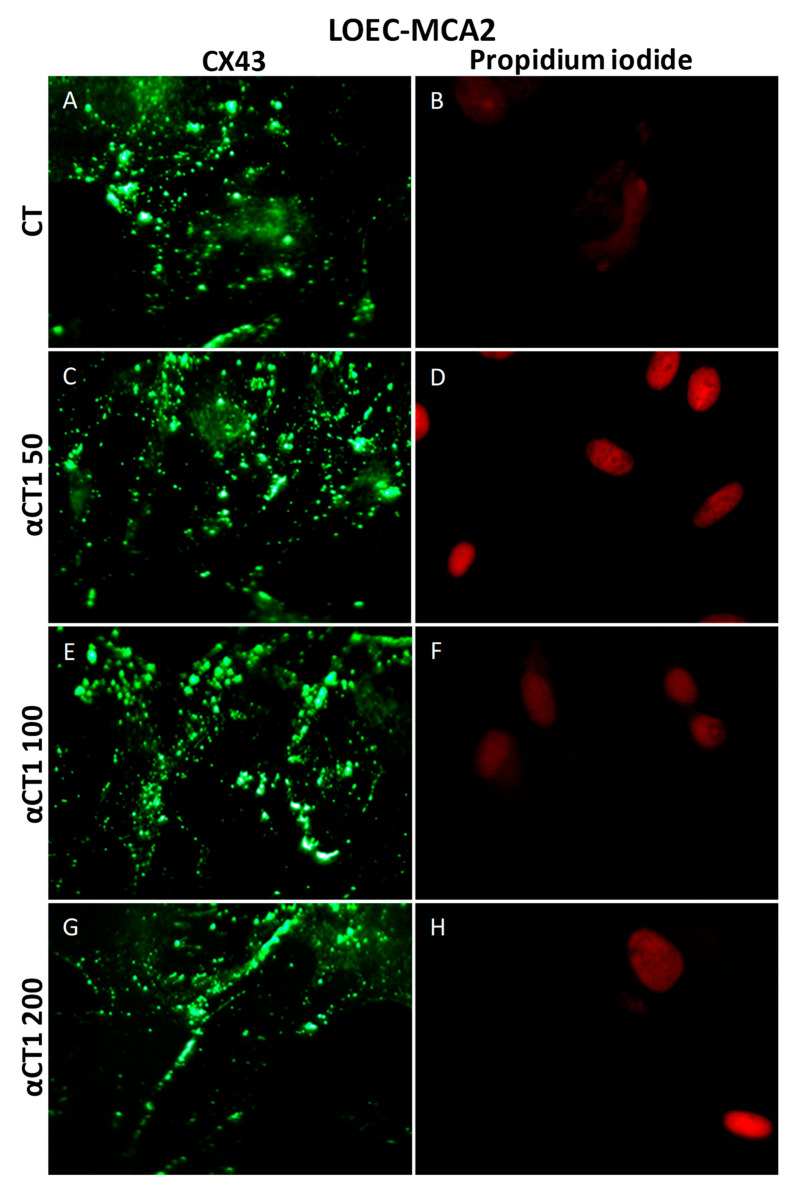
Immunofluorescence photomicrograph for CX43 in the canine mammary adenocarcinoma lineage LOEC-MCA2. In (**G**), the strong positivity of Cx43 in the cell membrane can be observed; (**A**,**C**,**E**,**G**) show immunofluorescence positivity of Cx43 in cell membranes; (**B**,**D**,**F**,**H**) show cell nuclei stained with propidium iodide. 40× objective.

In canine mammary adenocarcinoma cells MCA2, the same effect was observed: connexin 43 was localized in the cytoplasm in control cells, and in αCT1-peptide-treated cells, there was an increase in the Cx43 plaques in cell membranes (Figure 12).

**Figure 13:**
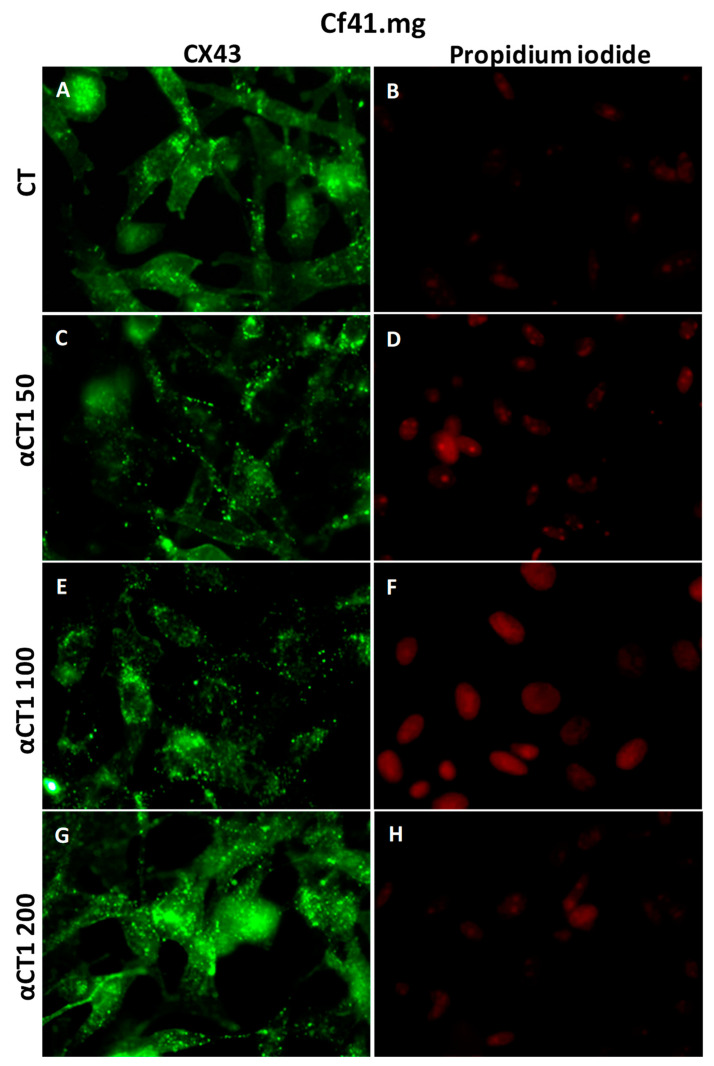
Immunofluorescence photomicrograph for CX43 in the canine mammary lineage CF41. More Cx43 spots in cytoplasm and membranes are seen with the highest concentration of αCT1 (**G**); (**A**,**C**,**E**,**G**) show immunofluorescence positivity of Cx43 in cell membranes; (**B**,**D**,**F**,**H**) show cell nuclei stained with propidium iodide. 40× objective.

CF41 cells presented a low expression of Cx43, mostly at the cytoplasm; however, when submitted to treatment with the αCT1 peptide at all concentrations, an increase in the Cx43 plaques in cell membranes was observed (Figure 13).

## 4. Discussion

Mammary neoplasms are the most prevalent type of neoplasm in humans, and their treatment, in advanced cases, is still challenging despite the high number of research projects found in the literature. The same information is true for canine mammary cancers. New treatments for both human and canine mammary cancers are needed; furthermore, preventive initiatives for mammary cancer should be urgently defined.

Gap junctions are communication junctions that are impaired in cancer cells. This information was reported by Loewenstein and Kanno in 1966 [9].

Since then, new therapies that aim to change the communication capacity of cancer cells have been tested, and few treatments based on natural substances have been found to increase cell–cell communication. Examples of these natural substances are green tea [62] and soybeans, which contain the Bowman–Birk inhibitor [60,63].

According to Nalewajska et al. [64], potential therapeutic strategies involving modulation of gap junctions or connexins in cancer can be categorized into at least six groups: (1) chemical compounds; (2) mimetic peptides; (3) inhibitors; (4) antibodies; (5) viral carrying therapies; and (6) nanocarriers. A common observation is that connexins or GJIC are typically downregulated in cancer cells, particularly in early-stage cancers. Restoring GJIC and increasing connexin expression have been suggested as potential therapeutic approaches.

An intriguing and promising approach to targeting gap junctions and connexins therapeutically involves the use of connexin mimetic peptides and specific antibodies to regulate gap junction (GJ) function and connexin expression. One such peptide is αCT1, which consists of 25 amino acids and is designed to mimic the carboxyl terminus of Cx43. At the molecular level, αCT1 hampers the activity of Cx43 hemichannels by inducing their sequestration from the perinexus region surrounding gap junctions. This action reduces hemichannel density and availability for activation within the cell membrane. While αCT1 is anticipated to enhance scarring outcomes following surgery in humans, it is also believed to possess anticancer properties.

The impact of the αCT1 peptide on human mammary cells has been previously documented. Grek et al. [1] demonstrated that therapeutic modulation of Cx43 by αCT1 preserves Cx43 at gap junction sites along the cell–cell membrane borders of breast cancer cells and enhances gap junction activity in functional assays. Furthermore, the heightened Cx43 gap junctional activity induced by αCT1 treatment inhibits the proliferation or survival of breast cancer cells while having no effect on non-transformed MCF10A cells.

This study evaluated the effects of αCT1 peptide in seven canine mammary cell lines: one normal epithelial mammary cell line, two adenomas, and four mammary adenocarcinomas. The results showed that the normal mammary cell line is resistant to treatment with αCT1, which is compatible with the study by Grek et al. [1]. One of the adenoma cell lines (myoepithelial adenoma) was also resistant to treatment with αCT1, although the other (mixed adenoma) was susceptible to treatment, mostly at 72 h after treatment.

The four canine adenocarcinoma cell lines differ regarding the susceptibility to the treatment with αCT1 peptide. One canine mammary cell line (LOEC-MCA3) was resistant to treatment; therefore, no reduction in cell viability was observed after treatment with αCT1 peptide for 48, 72, or 96 h. Three cell lines, canine mixed adenocarcinoma (LOEC-MCA1), canine complex adenocarcinoma (LOEC-MCA2), and commercial canine mammary adenocarcinoma cell line CF41, were susceptible to treatment with αCT1. The viability of the commercial cell line CF41 was reduced at 48, 72, and 96 h after treatment with αCT1 peptide. In most of the mammary cells used in this study, a strong positivity for Cx43 was seen in the membranes 48 h after the treatment with αCT1 peptide.

The design of αCT1 aims to redirect uncoupled Cx43 hemichannels into gap junctions, leading to decreased turnover and increased aggregation of gap junctions, all while maintaining Cx43 protein levels unaffected. Considering our immunofluorescence assay findings, it appears that αCT1 may have an effect on cell membrane Cx43 activity, although we have not examined the mechanism involved. In the presence of the αCT1 peptide, some cell lines had their growth inhibited, as seen in the MTT assay. In normal canine mammary cells, the αCT1 peptide does not have an inhibitory effect on their growth; this is due to the maintenance of plaques and communication junctions in these cells, preserving the normal epithelial cells. One among two adenomas and two among four adenocarcinomas were sensitive and presented differential effects on the viability of canine mammary neoplastic epithelial cells. αCT1 effects may be seen mostly at 72 and/or 96 h post-treatment. Treatment with αCT1 peptide redirects connexins 43 to the cell membrane in an attempt to increase cell communication, as seen in immunofluorescence. αCT1 may be considered in therapeutic protocols for the treatment of canine mammary cancers.

## 5. Conclusions

In conclusion, this study demonstrates the potential of αCT1 peptide in treating canine mammary tumors under specific conditions. It decreased benign and malignant neoplastic canine mammary cell viability while keeping unaltered the normal canine mammary epithelial cells. Despite revealing its efficacy, the exact mechanisms influencing cancer cell viability remain unknown. Further experiments are essential to unravel the therapeutic effects of αCT1, paving the way for its consideration in therapeutic protocols for canine mammary cancers.

## Figures and Tables

**Table 1:** Canine mammary cell lines and their origins.

Lineage	Breed	Age (Years)	Neoplasm	Number of Passages
LOEC-NMG	Labrador Retriever	10	Normal canine mammary gland	P10
LOEC-MCA1	Yorkshire	7	Canine mixed mammary adenocarcinoma	P8
LOEC-MCA2	Teckel	8	Canine complex mammary adenocarcinoma	P11
LOEC-MCA3	Labrador Retriever	10	Canine mammary adenocarcinoma	P9
LOEC-MAd1	German Shepherd	10	Canine mixed mammary adenoma	P9
LOEC-MAd2	Dachshund	13	Canine myoepithelial mammary adenoma	P10
CF41.mg	Beagle	10	Canine mammary adenocarcinoma	P32

**Table 2:** Culture media used for each cell line.

Canine Mammary Cell Lines	Growing Conditions
LOEC-NMG, LOEC-MCA1, LOEC-MCA2, LOEC-MCA3, LOEC-MAd1, LOEC-MAd2	Advanced DMEM, HEPES (10 mM), L-glutamine (2 mM), 1% antibiotic–antimycotic, cholera toxin (10 ng/mL), 1% MEGS (mammary epithelial growth supplement), and 0.5% SFB
CF41.mg (commercial canine mammary tumor cell line)	DMEM, 10% fetal bovine serum, and L-glutamine (2 mM)

**Table 3:** Summary of the results after 48, 72, and 96 h of treatment with ACT1 peptide. Asterisks represent the significance level obtained with the ANOVA statistical test (* *p* < 0.05, ** *p* < 0.01, and *** *p* < 0.001). All the significant results indicate decreases in cell viability.

Treatment 48 h	LOEC-NMG	LOEC-MAd1	LOEC-MAd2	LOEC-MCA1	LOEC-MCA2	LOEC-MCA3	CF41.mg
αCT1 25 µM							
αCT1 50 µM							
αCT1 100 µM							*
αCT1 150 µM							
αCT1 200 µM							
αCT1 250 µM							
αCT1 300 µM							
**Treatment 72 h**	**LOEC-NMG**	**LOEC-MAd1**	**LOEC-MAd2**	**LOEC-MCA1**	**LOEC-MCA2**	**LOEC-MCA3**	**CF41.mg**
αCT1 25 µM		*					
αCT1 50 µM		*					***
αCT1 100 µM	*	*					***
αCT1 150 µM					**		***
αCT1 200 µM		*					***
αCT1 250 µM		***					***
αCT1 300 µM		*					***
**Treatment 96 h**	**LOEC-NMG**	**LOEC-MAd1**	**LOEC-MAd2**	**LOEC-MCA1**	**LOEC-MCA2**	**LOEC-MCA3**	**CF41.mg**
αCT1 25 µM				***			*
αCT1 50 µM				***			
αCT1 100 µM				***			*
αCT1 150 µM		*		***			*
αCT1 200 µM				***			*
αCT1 250 µM			*	***			
αCT1 300 µM				***			*

## Data Availability

The data that support the findings of this study are available from the corresponding author upon reasonable request.
